# Sunderland Infirmary

**Published:** 1893-02-11

**Authors:** 


### THE INSTITUTIONAL WORKSHOP.

### WITHIN THE HOSPITALS.

### SUNDERLAND INFIRMARY.

BY OUR SPECIAL COMMISSIONER.

This institution was described to us by the superintendent of a hospital in a neighbouring town as the jewel of northern medical institutions. Certainly no pains have been spared to make it pleasing to the visitor, and here is to be seen, on the whole, as good an interior effect, so far as decoration is concerned, as will be met with in most hospitals at the present day. The institution, which is on the pavilion principle, has been built in blocks from time to time, and those who have undertaken to raise the funds for each new wing have surpassed their predecessors in taste, and so have secured a result which taken in detail is remarkable and taken as a whole certainly most satisfactory.

The number of children admitted to treatment at the Sunderland Infirmary is larger, we believe, than that in any other institution of the kind. The only doubt which crossed our mind was the fear that too much bricks and mortar was crowded on to the site. This point does not at any rate press at the moment, seeing that the pavilions are only of two storeys, and that the hospital is a new one. Speaking as a whole, the institution presents few special features. It is a good type of a modern pavilion hospital. The management is good, and the enthusiasm which the institution excites amongst all classes of inhabitants in Sunderland must be in every way beneficial. The committee are evidently anxious, from the just pride they take in the hospital, to make it as comfortable in every department as possible.

FINANCE.

The Sunderland Infirmary is blessed with a secretary whose energy and success as a raiser of income has probably never been surpassed. He was one of the first to organise a movement amongst the working classes in support of the infirmary. For some years the increase in the workmen's subscriptions has been steady, rising from £3,784 in 1889 to £4,541 in 1891, an evidence of the endeavour to recognise the special claims which the hospital urges upon them in a spirit of liberality. The effect upon the income of the workmen's subscriptions will be apparent when we state that at the present time it amounts to much more than double the sum received from the general public. It is a remarkable feather in Mr. Robinson's cap that the total subscriptions have risen from £2,852 in 1879 to £8,071 in 1891. The Hospital Saturday collections showed a steady increase from 1888 to 1891. In 1892 they fell off, however, from some cause not explained. This institution has not yet begun to receive any large bequests, but there is reason to hope that in due course it will obtain its share from this most fruitful source.

According to the latest statements 2,063 in-patients and 2,649 out-patients were admitted to treatment in the course of the year. Of the 2,063 in-patients, 1,206 were surgical and 857 medical, and amongst both classes were distributed 569 children. It is satisfactory to note that the number of out-patients at this hospital shows a slight decrease. The average number of beds occupied is between 180 and 185. In-patients usually remain under treatment for about 31 days, or, in the case of children, for 38·9 days. The average cost per bed occupied for treatment, nursing, and maintenance, is stated in the report to be under £44, or inclusive of all expenditure £46.

NURSING.

The nursing of this institution is in the hands of the Tottenham Sisters. These Sisters are an English offshoot of Dr. Laseron's institution at Kaiserwerth, where Miss Nightingale was trained. According to a paper that has been circulated, there are eighteen Sisters engaged in Sunderland, although we understood at the date of our visit that they did not exceed half that number. The managing Sister takes probationer nurses for training in the Infirmary. After a month's trial, if approved, they receive in addition to uniform, board and lodging, £10 for the first year, £16 for the second, and £18 for the third year. Probationers get fourteen days holiday, nurses twenty-one. Any probationer nurse breaking her three years' engagement, at the end of which time if she passes her examination she is entitled to her certificate, has to pay a fine of £6 and give three months' notice. Once certificated, nurses receive £22, advancing to a maximum of £26 per annum. Nurses are on duty from half-past seven in the morning till half-past eight in the evening, they being allowed two hours off duty during this time every alternate afternoon. The infirmary has accumulated a fund of £589 to provide the nucleus of a Nurses' Pension Fund. We were a little surprised that the managers and the Committee had not yet approached the Royal National Pension Fund for Nurses with the object of being relieved of all further liability for pensions to their nursing staff. They would find they could effect a saving of expenditure under this head by affiliating with the Pension Fund, and we have very little doubt that attention has only to be called to the matter to ensure their taking the necessary steps with a view to ascertaining how far such an arrangement could be made in the best interests of the infirmary and its nursing staff.

